# Why Biotrophs Can't Live Alone

**DOI:** 10.1371/journal.pbio.1001097

**Published:** 2011-07-05

**Authors:** Robin Meadows

**Affiliations:** Freelance Science Writer, Fairfield, California, United States of America

**Figure pbio-1001097-g001:**
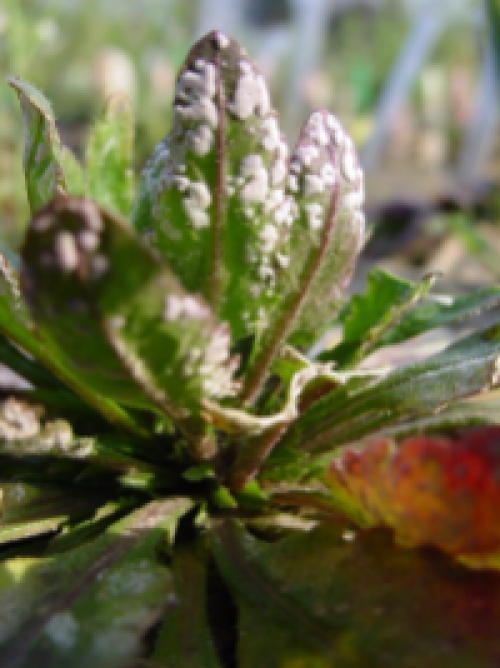
White blister rust of *Arabidopsis thaliana*, caused by the obligate biotroph oomycete *Albugo laibachii*, found in a field plot in Norwich, and later single-spore purified and sequenced.

[Fig pbio-1001097-g001]At first glance, diatoms, malaria parasites, and fungus-like plant pathogens called oomycetes look wildly different. But these organisms all have something in common: they belong to a group called the Chromalveolata that contains genes from algae. This diversity of lifestyles within a single group presents an opportunity to learn how the various strategies evolved. For example, oomycetes include both necrotrophs, which feed on dead plant tissue, and obligate biotrophs, which require living hosts. In addition, biotrophy evolved independently in two groups of oomycetes in the same lineage, the white rusts such as *Albugo laibachii* and the downy mildews such as the Irish potato famine pathogen *Phytophthora infestans* and *Hyaloperonospora arabidopsidis*, which infects the plant *Arabidopsis thaliana*.

Recent research on *H. arabidopsidis* has linked biotrophy to massive gene loss in biosynthetic metabolic pathways. This makes sense because there is less selection to synthesize products when the host provides them. Biotrophy is also thought to result from gaining ways to circumvent host defenses. However, the evolution and molecular mechanisms of this lifestyle are poorly understood. In this issue of *PLoS Biology*, Kemen, Jones, and colleagues confirm that obligate biotrophy entails pathway loss in oomycetes, and report a new class of molecules that suppress host plant defenses.

First, the researchers sequenced the genome of the white rust *A. laibachii* that, like the downy mildew *H. arabidopsidis*, is an obligate biotroph and parasitizes *A. thaliana*. Then they identified *A. laibachii* genes that code for proteins. This entailed extracting complementary DNA (which is made from the messenger RNAs that are translated into proteins) from *A. thaliana* leaves infected with *A. laibachii*, and then matching it to this white rust's genome sequences. The researchers also assigned *A. laibachii* genes to metabolic pathways using computational and manual prediction strategies.

To investigate the origins of biotrophy, the researchers compared genes from the two biotrophs (*A. laibachii* and *H. arabidopsidis*) with those of a hemibiotroph (*Phytophthora infestans*) and a necrotroph (*Pythium ultimum*) in the same oomycete lineage. The two biotrophs were the least related, confirming that this lifestyle arose twice in this lineage. To identify gene losses linked to biotrophy, the researchers looked for genes that were present in the hemibiotroph and the necrotroph but missing in the biotrophs. Both biotrophs were missing genes in nitrogen and sulfur acquisition pathways. This is in keeping with the hypothesis that the evolution of biotrophy involves losing biosynthetic pathways.

Likewise, the findings validate the other hypothesized component of biotroph evolution, suppression of host defenses. Biotrophic oomycetes form structures called haustoria that take up nutrients from and counteract defenses by their hosts. Outgrowths from intercellular hyphae enter plant cells and differentiate into haustoria, creating an intimate interface between host and parasite plasma membranes. Plants defend against invasion partly with disease resistance proteins that can recognize pathogen molecules. However, pathogens counteract plant defenses with secreted proteins called effectors, and in oomycetes some effectors have sequence motifs such as “RxLR”.

Comparison of secreted proteins from two *A. laibachii* strains revealed a new class of effectors with a CHxC motif. The researchers validated the CHxC effectors with a translocation assay that confers avirulence, which involves fusing the N-terminus of potential effectors to a known avirulence protein. The findings showed that one of the new CHxC effectors conferred avirulence as effectively as a previously known class of *A. laibachii* effectors.

To develop a model of how biotrophy evolves, the researchers determined the molecular divergence of *A. laibachii* from other Chromalveolata species with a variety of lifestyles: the other biotroph (*H. arabidopsidis*), the hemibiotroph (*P. infestans*), and the necrotroph (*P. ultimum*) in the same oomycete lineage, as well as a diatom (*Thalassiosira pseudonana*) and the malaria parasite *Plasmodium falciparum*. The researchers then assessed gene and metabolic pathway gains and losses amongst these species. For example, all of the oomycetes that form haustoria have also lost the pathway to make thiamine. In addition, like the oomycete biotrophs, the malaria parasite has lost molybdopterin-requiring pathways. This parasite also forms a structure that functions like a haustorium, taking up nutrients from the host and delivering secreted proteins to suppress host defenses.

Based on the gene and metabolic pathway patterns amongst these Chromalveolata species of known relatedness, the researchers propose that the first step towards obligate biotrophy in oomycetes is suppressing host defenses. This facilitates haustoria formation, letting oomycetes get nutrients from their hosts and so ultimately leading to the loss of biosynthetic pathways. This pathway loss then results in absolute dependence on the host.

This works sheds light on the origins and mechanisms of biotrophy, revealing a new class of avirulence proteins that can nullify plant defenses, and supporting the hypothesis that this lifestyle results from a combination of gaining ways to overcome host defenses and losing the ability to make nutrients. Understanding how organisms become obligate biotrophs that depend on specific hosts could also help lead to protections against human parasites in the Chromalveolata, including the malaria parasite and *Toxoplasma gondii*, which causes toxoplasmosis and can be fatal to fetuses and people with compromised immune systems.

**Kemen E, Gardiner A, Schultz-Larsen T, Kemen AC, Balmuth AL, et al. (2011) Gene Gain and Loss during Evolution of Obligate Parasitism in the White Rust Pathogen of Arabidopsis. doi:10.1371/journal.pbio.1001094**

